# Incoherent dose-escalation in phase I trials using the escalation with overdose control approach

**DOI:** 10.1007/s00362-016-0790-7

**Published:** 2016-06-24

**Authors:** Graham M. Wheeler

**Affiliations:** 1 MRC Biostatistics Unit Hub for Trials Methodology Research, Cambridge Institute of Public Health, Cambridge CB2 0SR, UK

**Keywords:** Bayesian statistics, Dose-escalation, Adaptive designs, Maximum tolerated dose, Phase I trials, Coherence

## Abstract

A desirable property of any dose-escalation strategy for phase I oncology trials is coherence: if the previous patient experienced a toxicity, a higher dose is not recommended for the next patient; similarly, if the previous patient did not experience a toxicity, a lower dose is not recommended for the next patient. The escalation with overdose control (EWOC) approach is a model-based design that has been applied in practice, under which the dose assigned to the next patient is the one that, given all available data, has a posterior probability of exceeding the maximum tolerated dose equal to a pre-specified value known as the feasibility bound. Several methodological and applied publications have considered the EWOC approach with both feasibility bounds fixed and increasing throughout the trial. Whilst the EWOC approach with fixed feasibility bound has been proven to be coherent, some proposed methods of increasing the feasibility bound regardless of toxicity outcomes of patients can lead to incoherent dose-escalation. This paper formalises a proof that incoherent dose-escalation can occur if the feasibility bound is increased without consideration of preceding toxicity outcomes, and shows via simulation studies that only small increases in the feasibility bound are required for incoherent dose-escalations to occur.

## 1 Introduction

Phase I clinical trials mark the first experimentation of a new drug in a human population. For cytotoxic anti-cancer drugs, the aim of a phase I trial is to gradually adapt the dose level of the drug given to patients in order to identify the Maximum Tolerated Dose (MTD), defined as the largest dose that leads to unacceptable toxicity in a target proportion, *θ*, of patients (Babb and Rogatko 2004). The rationale for targeting such a dose is based on the assumption that higher doses will be more effective, yet more toxic (Green et al. 2003), and that toxicity is tolerable for optimal anti-tumour activity (Babb et al. 1998). Toxicities are graded according to the National Cancer Institute’s Common Terminology Criteria for Adverse Events (NCI CTCAE) (NCI 2009), and are usually reduced to a single binary outcome, which denotes whether a Dose-Limiting Toxicity (DLT) has occurred or not (Le Tourneau et al. 2009). Therefore, for a pre-specified Target Toxicity Level (TTL) of *θ*, the definition of the MTD can be expressed mathematically as

(1) ℙ(DLT|dose=MTD)=θ.

Since an unknown portion of the dose range will be too toxic for patients, a *dose-escalation* study is conducted, rather than randomly allocating patients over discrete dose levels and then estimating the MTD (Demirhan and Demirhan 2015). Furthermore, sample sizes in phase I oncology trials are often very small, which means that multiple testing procedures that incorporate dose-toxicity orders are not particularly useful (Pigeot 2000). To avoid these issues, several Bayesian adaptive methods, which sequentially recommend dose adaptations and borrow information from lower dose levels and prior beliefs, have been proposed for conducting dose-escalation studies and estimating the MTD (O’Quigley et al. 1990; Babb et al. 1998; Cheung and Chappell 2000). The escalation with overdose control (EWOC) approach (Babb et al. 1998) is a Bayesian adaptive design that reduces the risk of overdosing patients by choosing doses with a posterior probability of being above the true MTD equal to some value known as a *feasibility bound*. The feasibility bound, denoted as *α*, controls how conservative dose-escalation during the trial is and was originally suggested to be fixed throughout the trial. Several publications (Babb and Rogatko 2001; Cheng et al. 2004; Tighiouart and Rogatko 2010) describe trials where *α* increases during the trial so that eventually dose-selection is based on the posterior median of the MTD distribution; at this point the posterior probability of dosing above the true MTD is identical to dosing below the true MTD. Whilst such a design provides improved operating characteristics relative to the EWOC approach with a fixed feasibility bound (Chu et al. 2009), there is no guarantee of *coherent* dose-escalation (Cheung 2005; Tighiouart and Rogatko 2010; Cheung 2011) that is, dose escalation may be recommended despite having observed a DLT in the previous patient.

This paper formalises a proof that incoherent dose-escalation can occur when the feasibility bound is increased after observing toxicity in a dose-escalation trial using the EWOC approach. Along with a new theoretical result, several simulation studies are conducted for a trial of 5-fluorouracil (5-FU) (Babb et al. 1998) using the EWOC approach to see which situations are more likely to yield incoherent dose escalations when the feasibility bound is increased during a trial. Recommendations for practical implementation of the EWOC approach with a varying feasibility bound are provided in the Discussion.

## 2 Escalation with overdose control

### 2.1 Overview

Let *Y_i_* be a binary random variable such that *Y_i_* = 1 if patient *i* experiences a DLT and *Y_i_* = 0 otherwise. For a dose range of interest, bounded below by *x*_min_ and above by *x*_max_, denote the probability of DLT for patient *i* at dose level *x* ∈ [*x*_min_, *x*_max_] by *π* (*x*; ***β***), where ***β*** is a parameter vector. Several structural forms for *π* (*x*; ***β***) have been proposed (Cheung 2011), but we shall only consider the two-parameter logistic model proposed in the original EWOC paper (Babb et al. 1998), i.e.

(2) $$\mathbb{P}\left(Y_{i}=1|\;\text{dose}=x\right)=\pi \left(x;\beta_{0},\beta_{1}\right)=\frac{\text{exp}\left(\beta_{0}+\beta_{1}x\right)}{1+\text{exp}\left(\beta_{0}+\beta_{1}x\right)},$$

where *β*_0_ and *β*_1_ are parameters to be estimated and *β*_1_ > 0 to ensure the assumption of monotonicity is satisfied (i.e. probability of DLT is non-decreasing with dose). Rearranging Eq. 2 using Eq. 1, the MTD, denoted as *γ*, can be written as

(3) $$\gamma =\frac{\text{logit}\left(\theta \right)-\beta_{0}}{\beta_{1}}.$$

Under the original EWOC approach, *π* (*x*; *β*_0_, *β*_1_) is expressed in terms of two clinically relevant parameters: the MTD *γ* (Eq. 3); and the probability of DLT at the lowest dose level to be used in the trial, denoted as *ρ*_0_, where

(4) $$\rho_{0}=\pi \left(x_{\text{min}};\beta_{0},\beta_{1}\right)=\frac{\text{exp}\left(\beta_{0}+\beta_{1}x_{\text{min}}\right)}{1+\text{exp}\left(\beta_{0}+\beta_{1}x_{\text{min}}\right)}.$$

We therefore write *π* (*x*; *β*_0_, *β*_1_) as *π* (*x*; *γ*, *ρ*_0_) and use a Bayesian updating procedure by placing prior distributions upon *γ* and *ρ*_0_ (Kadane et al. 1980); Babb et al. (1998) suggest a Uniform prior distribution for *γ* over the interval [*x*_min_, *x*_max_], and a Uniform prior distribution for *ρ*_0_ over the interval [0, *θ*], since *ρ*_0_ > *θ* implies that the MTD *γ* is lower than *x*_min_.

We condition all subsequent calculations on the event that *Y*_1_ = 0 (i.e. the first patient did not experience a DLT; if *Y*_1_ = 1, then it is recommended that the trial is suspended for safety concerns and the experimental dose range re-evaluated or the trial terminated (Babb et al. 1998; Tighiouart et al. 2005; Tighiouart and Rogatko 2010)). Given the set of trial data for *n* patients 𝒟_*n*_ = {(*x_i_*, *y_i_*) : *i* = 1,…, *n*}, where patient *i* received dose *x_i_* ∈ [*x*_min_, *x*_max_] and had outcome *y_i_* ∈ {0, 1}, the joint likelihood function for *γ* and *ρ*_0_ is

$$L\left(\gamma ,\rho_{0}|{𝒟}_{n}\right)=\prod_{i=1}^{n}\pi {\left(x_{i};\gamma ,\rho_{0}\right)}^{y_{i}}{\left[1-\pi \left(x_{i};\gamma ,\rho_{0}\right)\right]}^{1-y_{i}}.$$

For some joint prior *f* (*γ*, *ρ*_0_) on parameters *γ* and *ρ*_0_ (we assume the aforementioned independent Uniform priors; other frameworks are available (Tighiouart et al. 2005)), we obtain the joint posterior distribution *g* (*γ*, *ρ*_0_ | 𝒟*_n_*) via Bayes’ Theorem and hence the marginal posterior cumulative distribution function (CDF) for the MTD *γ* is

(5) $$H_{n}\left(\gamma^{\prime }\right)=\mathbb{P}\left(\gamma \leq \gamma^{\prime }|{𝒟}_{n}\right)=\int_{x_{\text{min}}}^{\gamma^{\prime }}\int_{0}^{\theta }g\left(\gamma ,\rho_{0}|{𝒟}_{n}\right)d\rho_{0}d\gamma .$$

Dose allocation for future patients is determined by selecting the 100*α*th percentile from the posterior MTD distribution, i.e. the dose for the (*n* + 1)th patient, denoted *x*_*n*+1_, is $x_{n+1}=H_{n}^{-1}\left(\alpha \right).$ The constant *α* is defined as the *feasibility bound* and governs the degree of conservatism present in the trial. The feasibility bound can be interpreted via a decision-theoretic loss function, which describes the relative preference of underdosing a patient compared to overdosing a patient. For some dose level *x* and MTD *γ*, the loss function for feasibility bound *α* is

(6) $$\text{Loss}\left(x,\gamma \right)=\{\begin{array}{ll} \alpha \left(\gamma -x\right) & \text{if}\;x\;\text{is}\;\text{an}\;\text{underdose},\;\text{i}.\text{e}.\;x\leq \gamma \\ \left(1-\alpha \right)\left(x-\gamma \right) & \text{if}\;x\;\text{is}\;\text{an}\;\text{overdose},\;\text{i}.\text{e}.x\geq \gamma . \end{array}$$

Equivalently, for any *δ* > 0, the loss incurred by overdosing a patient (with respect to the MTD *γ*) by *δ* units is $\frac{1-\alpha }{\alpha }$ times greater than underdosing a patient by *δ* units (Babb et al. 1998; Babb and Rogatko 2001). For *α* < 0.50, the loss function in Eq. 6 places a higher penalty on overdosing, whereas *α* = 0.50 penalises overdosing and underdosing equally severely. We only consider the loss function given in Eq. 6 for dose recommendations, though alternative myopic loss functions, or even balanced loss functions (if we wished to estimate the dose-toxicity relationship in full as well as identify the MTD) could be considered (Jozani et al. 2012).

### 2.2 Increasing the feasibility bound mid-trial

The idea of increasing the feasibility bound during the trial has been discussed (Babb and Rogatko 2001, 2004) and used in practice (Babb and Rogatko 2001; Cheng et al. 2004; Tighiouart and Rogatko 2010). At the beginning of the trial *α* is set to some minimal level strictly less than 0.50, so that the first patients that enter the trial are treated at safe doses with a high probability. As data are accrued, one can afford to be less conservative about dose-escalation, since the precision of the MTD distribution is increasing. To facilitate this, *α* can be gradually increased towards 0.50, at which point patients will be treated at the posterior median estimate of the MTD distribution. With respect to the loss function in Eq. 6, when *α* tends towards 0.50, the implication is that investigators become less concerned with underdosing relative to overdosing; when *α* = 0.50, the penalty for underdosing is identical to that of overdosing.

## 3 Coherence violations

For fixed *α* throughout the trial, the EWOC approach is coherent in escalation and de-escalation (Tighiouart and Rogatko 2010). We show that for increases in *α* after observing DLT outcomes, incoherent dose-escalation may occur.

### 3.1 Theoretical work

Let *H_n_*(*γ*) be the posterior CDF of the MTD parameter *γ*, as defined in Eq. 5. Define *α_n_* to be the value of *α* used to choose the dose *x_n_* ∈ [*x*_min_, *x*_max_] for patient *n*. Therefore $H_{n-1}\left(x_{n}\right)=\alpha_{n}\Leftrightarrow H_{n-1}^{-1}\left(\alpha_{n}\right)=x_{n}.$ First, we recall what it means to be *coherent in dose-escalation*.

**Definition 1** (*Coherent in dose-escalation*) Let *H_n_*(*x*) denote the posterior CDF of the MTD parameter *γ* given trial data for the first *n* ≥ 2 patients. Assume *H_n_*(*x*) is well-defined and infinitely differentiable on (*x*_min_, *x*_max_). A dose-escalation design is said to be coherent in dose-escalation if and only if *x*_*n*+1_ ≤ *x_n_* whenever *y_n_* = 1.

To show coherence in dose-escalation for the EWOC approach with fixed *α*, it is sufficient to show *H_n_*(*x*) ≥ *H*_*n*−1_(*x*) for all *x* ∈ [*x*_min_, *x*_max_] and *n* ≥ 2.

**Theorem 2** (Coherence of EWOC with fixed *α*) *For α_n_* = *α* ∈ [0, 1] *for all n, when* $y_{n}=1,H_{n}\left(t\right)\geq H_{n-1}\left(t\right)for\;all\;t\in \left[x_{\text{min}},x_{\text{max}}\right]\Leftrightarrow H_{n}^{-1}\left(\alpha \right)\leq H_{n-1}^{-1}\left(\alpha \right)\Leftrightarrow x_{n+1}\leq x_{n}.$

*Proof* See appendix of Tighiouart and Rogatko (2010) for proof.

We build upon this result to prove the possibility of incoherent dose-escalation when the feasibility bound is increased following a patient experiencing a DLT.

**Theorem 3** (Non-guarantee of coherence in escalation) *Assume that $H_{n}^{-1}\left(1\right)>x_{n},$ where *H_n_*(*x*) is as defined by Eq. 5 and Definition* 1. Then there exists some α* > *α_n_* *such that* $H_{n}^{-1}\left(\alpha^{*}\right)>H_{n-1}^{-1}\left(\alpha_{n}\right)$ *when* *y_n_* = 1.

*Proof* Both *H_n_*(*x*) and *H_n−1_*(*x*) are continuous and non-decreasing in *x*. By applying Theorem 2 and the Intermediate Value Theorem, and given $H_{n}^{-1}\left(1\right)>x_{n},$ (i.e. *x_n_* < inf {*x* : *H_n_*(*x*) = 1}), there exists some *α*′ ≥ *α_n_* that must give $H_{n}^{-1}\left(\alpha^{\prime }\right)=x_{n}=H_{n-1}^{-1}\left(\alpha_{n}\right).$ Furthermore, since *H_n_* is continuous and non-decreasing, $H_{n}^{-1}\left(\alpha^{*}\right)>H_{n-1}^{-1}\left(\alpha^{\prime }\right)$ for α* > *α*′, with equality existing if and only if lim_*t* →*x_n_*_ *H*′ (*t*) = ∞, which violates the assumption that *H_n_*(*x*) is infinitely differentiable on the interval (*x*_min_, *x*_max_). Therefore, given $H_{n}^{-1}\left(1\right)>x_{n},$ there exists an α* satisfying *α_n_* ≤ *α*′ < α* ≤ 1 such that $H_{n}^{-1}(\alpha *)>H_{n-1}^{-1}(\alpha_{n})$ when *y_n_* = 1.

Theorem 3 considers the case where $H_{n}^{-1}\left(1\right)>x_{n},$ otherwise we are in the case where $H_{n}^{-1}\left(1\right)<H_{n-1}^{-1}\left(\alpha_{n}\right)=x_{n},$ and incoherent escalation is impossible for all *α* ≥ *α_n_*. Whilst this is entirely plausible, Theorem 3 shows that there can still exist instances whereby incoherent dose-escalation may occur. We explore this with a practical example in Sect. 3.2.

### 3.2 Practical example

Consider the trial described by Babb et al. (1998) that used the EWOC approach to find the MTD of 5-fluorouracil (5-FU) when given in combination with 20 mg/m^2^ leucovorin and 0.5 mg/m^2^ topotecan to patients with malignant solid tumours. In this trial, *x*_min_ = 140, *x*_max_ = 425 and $\theta =\frac{1}{3}.$ The dose-toxicity model in Eq. 2 was used with *γ* ~ *U* [*x*_min_, *x*_max_] and *ρ*_0_ ~ *U* [0, *θ*] *a priori*, and *γ* and *ρ*_0_ independent. For this trial, *α* was fixed at 0.25 throughout. We simulate a trial of 40 patients, assuming the true MTD value (*γ*^True^) is 300 mg/m^2^ and the true probability of DLT at $x_{\text{min}}\left(\rho_{0}^{\text{True}}\right)$ is 0.08. We observe the minimum size difference between *α*_*n*+1_ and 0.25 required to generate an incoherent dose-escalation, had an increasing feasibility bound approach been implemented after patient *n*, via the following procedure:

1. Let *n* = 1 and *α* = 0.25. Dose patient 1 at *x*_1_ = *x*_min_ and let *Y*_1_ = 0 (otherwise trial does not proceed).
2. For 2 ≤ *n* ≤ 40:
  (a) Given trial data 𝒟_*n*−1_ = {*x*_1_, *y*_1_,…, *x*_*n*−1_, *y*_*n*−1_}, dose patient *n* at the dose recommended as per the standard EWOC approach $\left(x_{n}=H_{n-1}^{-1}\left(\alpha \right)\right)$ and set *y_n_* = 1.
  (b) Obtain the posterior CDF for *γ*, *H_n_*(*γ*).
  (c) Using *H_n_*(*γ*), find the minimum value of *α* ∈ 𝒜 = {0.26,…, 0.50} that gives an incoherent dose-escalation for patient *n* + 1, i.e.
    (7) $$\alpha_{n+1}^{\text{min}}=\text{min}\;\left\{\alpha \in 𝒜:H_{n}^{-1}\left(\alpha \right)>x_{n}\right\}.$$
  (d) Record $\alpha_{n+1}^{\text{min}}.$ and re-generate *Y_n_* from the Bernoulli distribution with probability $\pi \left(x_{n};\gamma^{\text{True}},\rho_{0}^{\text{True}}\right).$
  (e) Repeat steps a)-d) with updated sample size *n* ← *n* + 1 and updated filtration 𝒟_*n*_ ← {𝒟_*n*−1_, *x_n_*, *Y_n_* }.

Table 1 shows one simulated trial, with patient number, observed DLT outcome, dose given and minimum feasibility bound required to guarantee incoherent dose-escalation, should the DLT outcome of the previous patient actually be equal to 1. As more patients are recruited into the trial, the value of $\alpha_{n+1}^{\text{min}}$ tends to decrease. This is because as more data are accrued, the variance around *H_n_*(*γ*) decreases and new data provide smaller shifts in the position of *H*_*n*+1_(*γ*) relative to *H_n_*(*γ*). The same phenomenon will occur when strong prior distributions are placed on the model parameters (see Sect. 3.3) and therefore $\alpha_{n+1}^{\text{min}}$ for small *n* is more likely to be much lower than the figures presented in Table 1. Although this is only one simulated trial, increases in the feasibility bound by 0.04 or 0.05, which are increment sizes that have been used in actual trials (Tighiouart and Rogatko 2010), generate incoherent escalations in patients recruited into the trial later on. We now conduct several simulation studies to explore how the number of dose levels available and the strength of prior probability distributions affect the distribution of $\alpha_{n+1}^{\text{min}}.$

### 3.3 Simulation studies

**Fig. 1:**
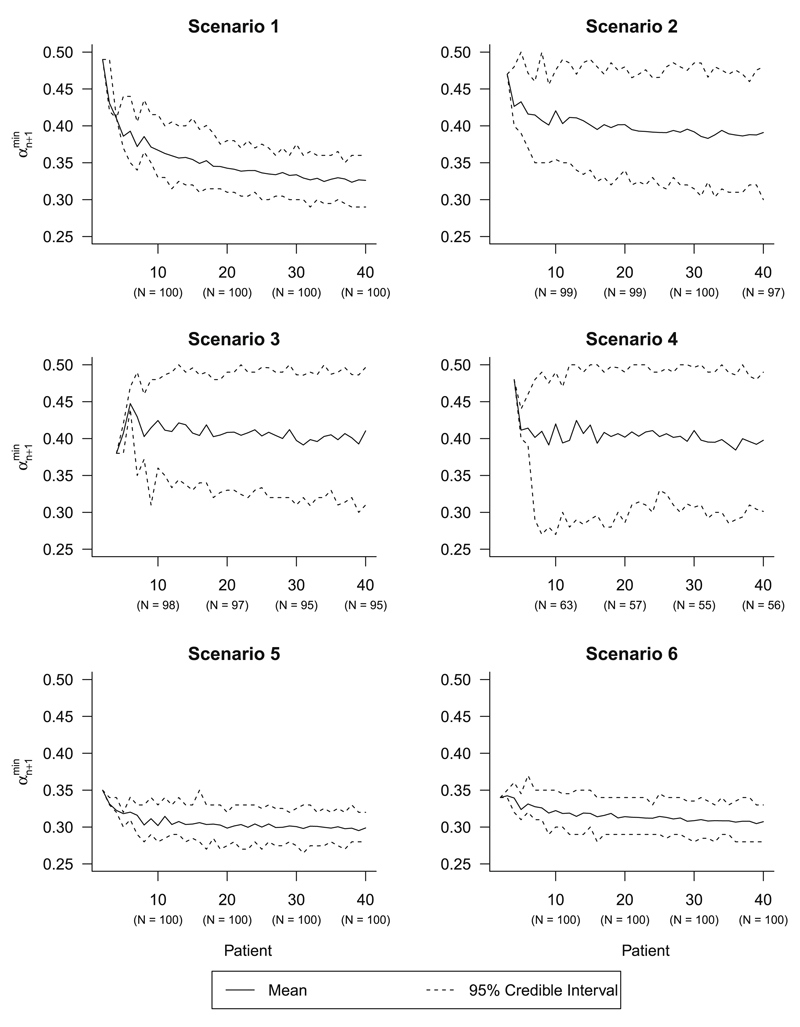
Distribution of minimum feasibility bounds causing incoherence ($\alpha_{n+1}^{\text{min}}$) over patient number for scenarios 1–6. Number of trials at patients 10, 20, 30 and 40 that yielded $\alpha_{n+1}^{\text{min}}$ within the interval [0*.*26*,* 0*.*50] shown in *parentheses*

To investigate the required increase in the feasibility bound to yield incoherent dose escalations, simulation studies for six different EWOC trial setups were conducted (Table 2). Scenario 1 is identical to the setup specified in Sect. 3.2, and scenarios 2, 3 and 4 are the same as scenario 1, but with discrete dose levels at different intervals. Scenarios 5 and 6 are the same as scenario 1, except the priors are specified differently; scenario 5 has skewed priors that place more weight on the MTD being at the lower end of the dose range, whereas scenario 6 is a strong prior that assumes the MTD is in the middle of the dose range. Both of these scenarios depend on Beta prior distributions that assume an effective sample size of 10 patients (calculated by summing the parameters of the Beta distribution). For each scenario, 100 trials were simulated using the same procedure specified in Sect. 3.2. Figure 1 shows the mean and 95% credible intervals for the distribution of $\alpha_{n+1}^{\text{min}}$ as the trial progresses for all six scenarios. Across scenarios 1, 2, 3 and 4, the mean trajectory of $\alpha_{n+1}^{\text{min}}$ over the trial differs depending on the number of dose levels; on average, larger increases in the feasibility bound are required to generate incoherent dose escalations as the number of available dose levels decreases. The 95% credible intervals are wider when fewer dose levels are available; this is because there are fewer instances when incoherent dose escalations arise when the feasibility bound can reach at most 0.50. Scenarios 2, 3 and 4, where 20, 16 and six evenly-spaced discrete dose levels are used respectively, show the mean of $\alpha_{n+1}^{\text{min}}$ to decrease to around 0.40 for most of the trial (scenario 4 95% CI (0.30, 0.49)), whereas scenario 1 shows a gradual mean decrease to 0.32 at patient 40 (95% CI (0.29, 0.36)). This means that upon observing a DLT after patient *n* in a trial, increases in the feasibility bound by 0.04 or 0.05 could be enough to provide an incoherent dose escalation for patient *n* + 1; in scenario 4 this occurs before patient 10, meaning that patients recruited at the start of the trial could be recommended an increase of at least 57 mg/m^2^ even after observing a DLT in the previous patient. Under scenarios 5 and 6, which used strong skewed and strong symmetric priors respectively, small increases in the feasibility bound are required to generate an incoherent dose-escalation even early on in a trial; at patient 10, the mean of $\alpha_{n+1}^{\text{min}}$ is 0.30 (95% CI (0.28, 0.33)). This is because the change in the posterior cumulative distribution function of the MTD after incorporating new data is much smaller when stronger priors are used.

## 4 Discussion

This paper formally outlines how incoherent dose-escalation can occur in phase I oncology trials when increasing the feasibility bound after observing toxicity under the EWOC approach. The example presented in Sect. 3.2 shows that even small increases in the feasibility bound can be enough to cause incoherent dose-escalation. The simulation studies presented in Sect. 3.3 also indicate that this is the case for different dose ranges and prior specifications. Interestingly, small changes in the feasibility bound could lead to incoherent dose-escalations being recommended early on in the trial, particularly if strong priors are used or few dose levels are considered. The key message is that incoherence can occur and that a design’s operating characteristics and chance of permitting incoherent escalation should be fully determined before an actual trial is conducted. Arbitrary increases in the feasibility bound as per the trials referenced in Sect. 2.2 are best avoided by escalating the feasibility bound only in the absence of DLTs, thus guaranteeing coherent dose-escalation and de-escalation. However, this should not exclude investigators from assessing how a trial design may perform for increases in the feasibility bound; large changes in *α_n_* increase the risk of patients experiencing severe toxicity. The approach for changing the feasibility bound should ideally be specified before the trial begins; ad hoc changes to the planned increases in the feasibility bound during the trial could result in a poor understanding of the design’s future behaviour, and on a practical level, require changes in the trial protocol to be made. Equally, one would be choosing the feasibility bound based on the dose that they wanted to use, rather than considering where it is on the MTD distribution. Before the trial, one may run simulation studies similar to those in Sect. 3.3 in order to determine how large increases in the feasibility bound might affect the dose-escalation behaviour of a trial design. This can be undertaken for trials with continuous or discrete doses, strong or weak priors, and can help clinicians determine when in the trial to reduce how conservative they wish to be in dose escalation. The results of this work show that in some scenarios, the feasibility bound need not increase by a lot before an incoherent escalation is observed, which suggests it is safer to increase the feasibility bound only in the absence of toxicity, whilst still converging to the MTD (Zacks et al. 1998). Bartroff and Lai (2011) have previously considered the frequency of coherence violations under the EWOC design with increasing feasibility bounds, yet focused on the ability of the model to recommend the correct MTD and other operating characteristics. Whilst designs with superior operating characteristics with respect to patient safety and accurate MTD estimation are to be encouraged in practice, ensuring that incoherent dose escalation is not possible should also be a priority to prevent unsafe dose escalations being recommended and reduce the risk of having to make unexpected changes to the design mid-trial. Even for approaches that converge to the true MTD, the fluctuation of the dose level around and above the true MTD means that incoherent escalations may occur at both low and high dose levels. Therefore, there is a risk of escalating the dose to a severely toxic level when the feasibility bound is increased after observing a DLT, and this can be from either a tolerable or intolerable dose.

It should be made explicit that this work is not a refutation of the EWOC approach, or indeed a call to prevent changing the feasibility bound mid-trial. Model-based adaptive designs for phase I trials, many of which have been shown to supersede the traditional 3 + 3 approach (Carter 1973; Storer 1989) have been carefully developed over the last 25 years (Le Tourneau et al. 2009), and much work has been done to increase their prevalence in clinical practice. The EWOC approach is a welcome addition to the family of model-based designs and increasing the feasibility bound during a trial is a sensible idea in order to escalate towards the true MTD faster than usual whilst mitigating the risk of overdosing patients. Whatever the choice of dose-escalation design, operating characteristics should be well-assessed and compared to other available approaches, and should be done so on a trial-by-trial basis.

## Figures and Tables

**Table 1:** Minimum value of feasibility bound $\alpha_{n+1}^{\text{min}}$ that leads to incoherent dose-escalation for patient *n* + 1 following *n* patients dosed under the original EWOC approach with fixed feasibility bound, assuming that patient *n* has a DLT

Patient (*n*)	DLT	Dose	$\alpha_{n+1}^{\text{min}}$	Patient (*n*)	DLT	Dose	$\alpha_{n+1}^{\text{min}}$
1	0	140	–	21	1	279	0.32
2	0	211	0.50	22	1	268	0.32
3	0	243	0.44	23	0	258	0.32
4	0	261	0.39	24	1	260	0.30
5	0	276	0.39	25	0	252	0.32
6	0	290	0.37	26	1	257	0.32
7	0	300	0.37	27	1	246	0.31
8	0	311	0.34	28	0	238	0.30
9	0	319	0.35	29	0	243	0.32
10	0	328	0.35	30	1	245	0.30
11	1	336	0.34	31	1	237	0.32
12	0	320	0.35	32	1	229	0.30
13	1	328	0.36	33	0	223	0.31
14	1	311	0.33	34	1	225	0.31
15	0	297	0.33	35	0	219	0.30
16	0	303	0.33	36	0	222	0.31
17	1	310	0.34	37	1	224	0.30
18	1	297	0.33	38	1	217	0.31
19	0	285	0.32	39	0	212	0.31
20	1	290	0.32	40	0	214	0.29

Actual DLT outcomes from simulated trial also given to show progression under EWOC approach with fixed feasibility bound

**Table 2:** Scenarios for simulation study, including number of dose levels and prior distributions

Scenario	Number of dose levels	*f* (*γ*)	*f* (*ρ*_0_)
1	286 (every 1 mg/m^2^)	*U* [*x*_min_ *, x*_max_]	*U* [0*,θ*]
2	20 (every 15 mg/m^2^)	*U* [*x*_min_ *, x*_max_]	*U* [0*,θ*]
3	16 (every 19 mg/m^2^)	*U* [*x*_min_ *, x*_max_]	*U* [0*,θ*]
4	6 (every 57 mg/m^2^)	*U* [*x*_min_ *, x*_max_]	*U* [0*,θ*]
5	286 (every 1 mg/m^2^)	*x*_min_ + (*x*_max_ – *x*_min_) × *Beta* [3, 7]	*θ* × *Beta* [7*,* 3]
6	286 (every 1 mg/m^2^)	*x*_min_ + (*x*_max_ – *x*_min_) × *Beta* [5, 5]	*θ* × *Beta* [5*,* 5]
